# Skeletal Morphogenesis and Anomalies in Gilthead Seabream: A Comprehensive Review

**DOI:** 10.3390/ijms242216030

**Published:** 2023-11-07

**Authors:** Kamel Mhalhel, Maria Levanti, Francesco Abbate, Rosaria Laurà, Maria Cristina Guerrera, Marialuisa Aragona, Caterina Porcino, Lidia Pansera, Mirea Sicari, Marzio Cometa, Marilena Briglia, Antonino Germanà, Giuseppe Montalbano

**Affiliations:** Zebrafish Neuromorphology Lab, Department of Veterinary Sciences, Via Palatucci Snc, University of Messina, 98168 Messina, Italy; kamel-mh@hotmail.fr (K.M.); mblevanti@unime.it (M.L.); franco.abbate@unime.it (F.A.); laurar@unime.it (R.L.); mguerrera@unime.it (M.C.G.); mlaragona@unime.it (M.A.); catporcino@unime.it (C.P.); lidia.pansera@studenti.unime.it (L.P.); mirea.sicari@studenti.unime.it (M.S.); marzio.cometa@unime.it (M.C.); marilena.briglia@unime.it (M.B.); gmontalbano@unime.it (G.M.)

**Keywords:** skeletal morphogenesis, gilthead seabream, ossification pattern, bone deformities, craniofacial anomalies, spinal deformities, genes, models

## Abstract

The gilthead seabream, one of the most important species in Mediterranean aquaculture, with an increasing status of exploitation in terms of production volume and aquafarming technologies, has become an important research topic over the years. The accumulation of knowledge from several studies conducted during recent decades on their functional and biological characteristics has significantly improved their aquacultural aspects, namely their reproductive success, survival, and growth. Despite the remarkable progress in the aquaculture industry, hatchery conditions are still far from ideal, resulting in frequent abnormalities at the beginning of intensive culture, entailing significant economic losses. Those deformities are induced during the embryonic and post-embryonic periods of life, and their development is still poorly understood. In the present review, we created a comprehensive synthesis that covers the various aspects of skeletal morphogenesis and anomalies in the gilthead seabream, highlighting the genetic, environmental, and nutritional factors contributing to bone deformities and emphasized the potential of the gilthead seabream as a model organism for understanding bone morphogenesis in both aquaculture and translational biological research. This review article addresses the existing lack in the literature regarding gilthead seabream bone deformities, as there are currently no comprehensive reviews on this subject.

## 1. Introduction

The gilthead seabream (*Sparus aurata*), one of the most important species in Mediterranean aquaculture, with an increasing status of exploitation in terms of production volume and aquafarming technologies, has become, with other teleost, an important research topic over the years [1,2,3,4,5,6]. The accumulation of new knowledge on aquaculture focusing on obtaining the most sustainable and profitable production and ensuring a high-quality product has significantly improved the seabream’s reproductive success, survival, and growth [7,8,9,10]. However, hatchery conditions are still far from ideal, resulting in frequent abnormalities, entailing significant economic losses [11,12,13,14,15]. Indeed, the quality of the fish depends on morpho-anatomic and organoleptic characteristics that should be as similar as possible to that of wild fish, which is the quality reference by the consumer [14]. Various skeletal abnormalities hindering the efficiency of the production cycle have been reported in many studies [7,11,14,16]. Other than downgrading the product’s image, these skeletal abnormalities can impact fish movement, physiological functions, and consequently, their health and survival.

Skeletal deformities in the gilthead seabream fry typically affect the cephalic region, namely the snout and the opercula, as well as the vertebral column and fins [12,14,15,17]. Those deformities are induced during the embryonic and post-embryonic stages, and their development is still poorly understood [14,18].

Numerous factors have been recognized as possible causes for deformities, ranging from nutritional, polluting sources to abiotic parameters, including hydrodynamic conditions, lighting, and stocking density [14,19,20].

Therefore, our main purpose in the present review was to summarize the existing research and to gather the current information available on gilthead seabream skeletal morphogenesis and provide valuable insights into the developmental processes and factors contributing to bone deformities in this species. Another aim of this review is to evaluate the potential of the gilthead seabream as a model organism for understanding bone morphogenesis in both aquaculture and translational biological research. This review article addresses the existing lack in the literature regarding gilthead seabream bone deformities, as there are currently no comprehensive reviews on this subject.

## 2. Skeletogenesis

Vertebrate skeletogenesis involves three main cell types: chondrocytes, osteoblasts, and osteoclasts [21]. The first two cell types secrete the extracellular matrix proteins of the cartilage and bone, respectively. In contrast, the third type produces matrix metalloproteinases, cathepsins, and tartrate-resistant acid phosphatase (TRAP), providing an acidic environment where the mineralized matrix is broken down [14,22]. In addition, a fourth type of cell known as osteocytes is involved in maintaining bone matrix, regulating bone formation and resorption, and acting as mechanical load sensors. Contrary to fish with cellular bone, teleosts, including the gilthead seabream, have derived skeletons composed only of acellular bone, a tissue without osteocytes in the mineralized matrix. The lack of osteocytes in acellular bone implies that bone remodeling is covered by other cell types [23]. Other than different types of cells, 65% of the dry mass of bone is made of hydroxyapatite salts, and the remaining part is a collagen fiber matrix [24]. Osteogenesis is ensured by a complex set of regulated molecular pathways involving signaling molecules, transcription factors, and extracellular matrix (ECM) constituents, among which alkaline phosphatase (ALP) or non-collagenous proteins, namely the matrix Gla protein (MGP) or osteocalcin (OC) [23]. Thus, the perturbation of those factors implicated in the control of bone development and homeostasis, as well as the incapacity of the self-regulating process to compensate for the stressful environmental conditions, induce the disruption of skeletogenesis and lead to the appearance of skeletal deformities [14,23].

Several studies have described skeletogenesis and the histological organization of skeletal tissues in the gilthead seabream, as well as the different typologies of skeletal deformities, in order to investigate the effects of different biotic and abiotic factors on the development of bone and the appearance of skeletal anomalies [14].

The skeleton ontogenesis revealed precedency to the onset of the elements that serve in feeding and respiration mechanisms (maxillary, Meckel’s cartilage, cartilaginous branchial arches) [12,25].

Studies on gilthead seabream meristic counts reported 24 vertebrae, 13 hemal arches, 23 neural arches, four pairs of parapophyses in the vertebral column region, five hypurals, one parahypural arch, three epurals in the caudal fin complex region beside all structures composing the cephalic skeleton [12,25,26].

### 2.1. The Ossification Pattern

Many research groups investigated the ossification state in farmed gilthead sea-bream larvae using acid-free double stains. During teleost embryogenesis, cartilaginous structures are the first element of the skeleton to form, which becomes bone after mineralization [17,27]. Our previous study demonstrated that this pattern of development was valid for gilthead seabream larvae [12].

**Figure 1:**
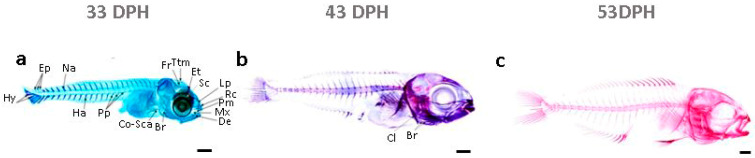
The ossification pattern of gilthead seabream larvae (**a**) at 33 DPH, (**b**) at 43 DPH, (**c**) at 53 DPH. Double-stained larvae showed alician blue-stained cartilaginous structures and alizarin red-stained bony structures. Fr, frontal; Ttm, taenia tecti medialis; Et, epiphysial tectum; Sc, sclerotic; Lp, lamina precerebralis; Rc, rostral cartilage; Pm, premaxillary; Mx, maxillary; De, dentary; Br, branchiostegal rays; Cl, cleithrum; Co-Sca, coraco scapular cartilage; Pp, parapophyse; Ha, hemal arches; Hy, hypural; Ep, epural; Na, neural arches. Scale bar: 0.5 mm. Schematic representation adapted from [12].

Figure 1 shows the chronology of ossification in the gilthead seabream between 33 and 53 days post-hatching (DPH) [12]. Until 33 DPH, the double staining revealed only cartilaginous structures, including epiphysial tectum, sclerotic, lamina precerebralis, rostral cartilage, premaxillary, maxillary, dentary, branchiostegal rays, cleithrum, and coraco scapular cartilage in the cranial region (Figure 1a). At 43 DPH, all cartilaginous structures earned volume, and the ossification began in the head region’s dentary, maxillary, and opercular complex (Figure 1b). Later, a saltatory ossification process was introduced on the vertebral column on centra 1–4, 8–10, and 14–19 (Figure 1b). The accomplishment of the ossification process occurred at 53 DPH when alizarin red staining had preeminence over the larva (Figure 1c).

### 2.2. The Genetics of Skeletogenesis

Skeletogenesis is a complex process encompassing skeleton patterning, cell differentiation, and cell function, during which the skeleton develops under the precise guidance of genetic programming, leading to the normal anatomy that provides support and protection for the internal organs [28]. It has been extensively described in several marine fish species [12,19,20], but gene expression patterns have been partially characterized in the gilthead seabream [29].

The expression of specific genes underlying the gilthead seabream’ cell proliferation and differentiation processes in association with ossification and bone remodeling and maintaining integrity was the aim of an in vitro study conducted by Tiago et al. using the VSa13 (pre-chondrocyte) and VSa16 (pre-osteoblast) bone-derived cell lines [30].

Global analysis (GO) of gene expression during in vitro mineralization of VSa13 and VSa16 identified 4223 and 4147 genes, respectively, differentially expressed. Among these, 3011 and 3049 up-regulated genes and 1212 and 1098 down-regulated genes were identified from VSa13 and VSa16, respectively. Those genes were classified according to their putative gene ontology and the up-regulated genes were principally associated with metabolism transport, matrix/membrane, and signaling, while down-regulated genes were associated with calcium binding, transport, and signaling [30].

In order to investigate genes involved in ECM mineralization, Tiago et al. exposed dividing and mineralizing VSa13 cells to vanadate, an ultra-trace element with anti-mineralogenic effect, and analyzed global gene expression. Thus, the genes oppositely regulated during in vitro mineralization and upon vanadate exposition would most likely be important for the differentiation/mineralization process. These genes and their respective global analysis categories are listed in Table 1 and have been classified according to FCM (control versus mineralization) and FCMV (mineralization versus mineralization + vanadate) ratio [30].

Comparative analysis of expression data from fish and mammalian cell systems reported that genes behind mechanisms of in vitro mineralization are conserved throughout vertebrate evolution and across cell types; thus, the same type of genes and, in some cases, the same orthologs were found to fulfill the same function [30,31].

## 3. Bone Deformities

In the last two decades, the gilthead seabream aquaculture industry has experienced a rapid development with impressive progress in rearing techniques, disease control, nutrition, and industrial hatcheries knowledge. As a maximal yield in growth success may come into reach, several problems arise with respect to the overall quality of the larvae and subsequent juvenile fish. The quality of the fish depends on morpho-anatomic and organoleptic characteristics that should be as similar as possible to that of wild fish, which is the quality reference by the consumer [14]. Various morphological abnormalities induced during the embryonic and post-embryonic periods of life, hindering the efficiency of the production cycle, have been reported in many studies [7,11,14,16]. These deformities, affecting as much as 80% of the fingerling production, cause an enormous economic slump in the industry, affecting the survival rates, growth, biological performance, the quality of the reared fish, the consumers’ overall perception of fish, and thus the cost-efficiency of marine fish aquaculture [17,32]. A significantly higher prevalence of anatomical abnormalities may be observed in gilthead seabream produced in intensive aquaculture than in wild-caught animals [14].

Despite the improvement in the rearing techniques, hatchery conditions in aquaculture farms are still far from ideal. Thus, the most frequent abnormalities are recorded at the beginning of gilthead seabream intensive culture, during embryonic and larval periods, long before osteological deformities are externally visible [14,33,34].

Skeletal deformities will fully develop to be visually identified only when fish are over 0.5 g in size and the affected batches have to be screened. Animals that carry the deformities must be detected and eliminated immediately since they will compete for food and space with healthy fish [35].

Skeletal deformities in gilthead seabream fry typically affect the cephalic region (snout and opercula), showing a deformed upper and lower jaw in a variety of shapes as well as operculum complex deformities affecting its bone series, leaving part of the gills exposed; the threshold traits of the vertebral column (vertebrae, neural, and hemal spin) as well as the meristic characteristics of the fin, especially the caudal fin complex deformities and saddleback syndrome [12,14,15,17,33,35].

Skeletal deformities and growth rates in the gilthead seabream have been the subject of many research studies. Some authors have associated the skeletal deformities with a defect in the inflation of the swim bladder [36]; others have attributed it to a dietary deficiency of fatty acids [37] and vitamins [38] as well as to the larval rearing systems [39] and rearing condition [40].

### 3.1. The Opercular Complex Deformities

**Figure 2:**
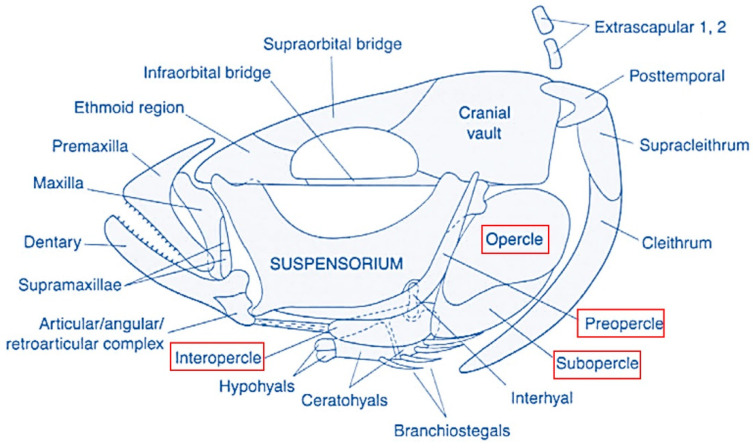
The teleostean operculum complex organization. Schematic representation adapted from [41].

The operculum is a hard, plate-like bony flap, made of opercles, subopercles, and interopercles, which overlie an opercular membrane forming together a gill cover or bony operculum protecting the orobranchial chamber. Below the opercular series, the branchiostegals are arranged in series with the sub- and interopercles and are attached proximally to the ventral face of the hyoid bar. The branchiostegal rays and membranes play only a passive role in the abduction and adduction of the branchial cavity and seem to serve primarily as a ventral-sealing valve (Figure 2) [41].

Among cephalic malformations, anomalies of the opercular complex are the most common and evident external abnormality in different fish species, especially in reared gilthead seabream, affecting up to 80% of the population [11,17,33,42].

**Figure 3:**
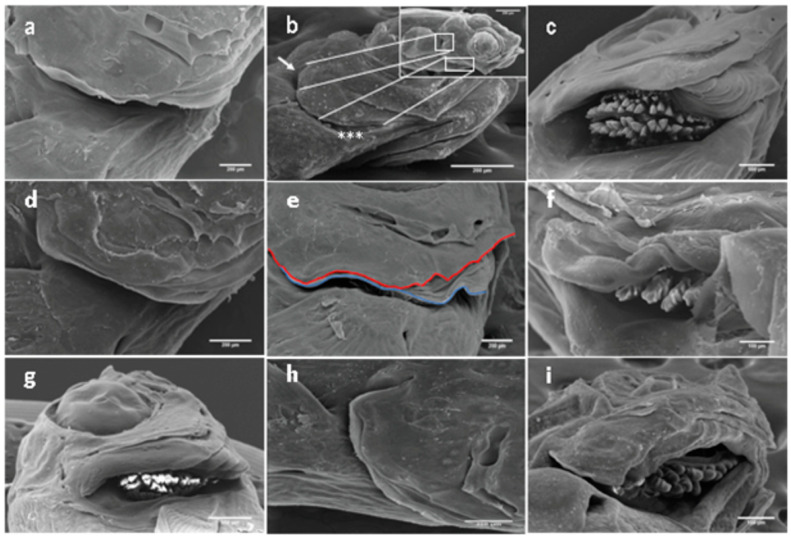
Different forms of the opercular complex abnormalities in gilthead seabream larvae aged 13–53 DPH. (**a**) Hypoplastic operculum, (**b**) reduced opercle (white arrows) and lack of interopercle (asterisks), (**c**) folded opercular bones toward the gills chamber leading to the exposition of gill arches, (**d**) outside folded operculum, (**e**) wave-like gill caver (red line) modulating a branchiostegal membrane (blue line), (**f**) spring-like gill caver with exposed gill arches, (**g**) combined shortened-folded operculum, (**h**) hyperplastic branchiostegal membrane, (**i**) folded branchiostegal rays in the gill chamber. Scale bars: 200 µm. Figure is adapted from Mhalhel et al. [12].

This malformation affecting the bone series of the operculum complex and the branchiostegal membrane consists of a reduction in the protecting wall for the orobranchial chamber on different levels, with references to a reduction or even lack of one of the various operculum bones (Figure 3a,b). Other types of anomalies are attributed to the inside or outside folding of one or more opercular bones (Figure 3c,d) or a combined shortened-folded operculum (Figure 3g). Moreover, we have detected for the first time new forms of operculum deformities, including wave-like (Figure 3e) and spring-like (Figure 3f) gill covers, as well as the lack of apposition of the branchiostegal membrane to the epithelium at the terminal edge of the branchial cavity generated by different degrees of hyperplasia (Figure 3h), and folded branchiostegal rays in the gill chamber (Figure 3i) [12].

Those operculum abnormalities are often associated with different degrees of malformation of the surrounding cranial region and exposure of the gills [7,33]. They may affect one or both opercula; however, the prevalence of monolateral ones is higher for bilateral ones. Until the last decade, the moment of apparition has not been precisely detected but was generally thought to be during the weaning and/or the pre-growing phase [7,33]. Still, its premonitory symptom and morphoanatomical description remain incomplete. In our study published in 2020, the first sign of opercular complex disorders was detected in 13 DPH larvae, a long time before the ossification process took place and earlier than the timing recorded by Galeotti et al. [12,17]. The wide variability in opercular complex deformity typologies hampered the founding of specific patterns of deformities, like those defined by Berlado et al. [43].

Moreover, the frequency of opercular anomalies on 13 to 83 DPH larvae ranged between 9.7% and 21.3%, as reported by Mhalhel et al. [12], which fits within the incidence scale extending from 6.3% to 43.2%, registered in prior studies conducted over the past two decades [12,14,43]. Those variances may be attributed to the different rearing systems and conditions [14,39] and to the accumulation of new knowledge in aquaculture [12]. Although the image and market value of the final product is impaired, opercular anomalies affect the breathing process during water intake and discharge, reduce resistance to environmental stress, especially reduced oxygen levels, and indirectly predispose the fish to gill diseases and bacterial infections [7,11,17].

Over the years, biotic, abiotic, physiological, xenobiotic, nutritional, and rearing factors have been incriminated as deterministic causes [33,43]. However, etiological knowledge is still insufficient for the elaboration of a Cartesian experimental hypothesis concerning the predisposing causes of apparition.

### 3.2. Other Cranial Deformities

According to previous studies, about 35–40% of the produced gilthead seabream had head deformities [7,12]. Various head deformations were associated with deoperculation, including a forward shift in the caudal margin of the opercular and subopercular, and the upward shift of the interopercle, the infraorbital, and shortening of the neurocranium and preorbital region [7].

**Figure 4:**
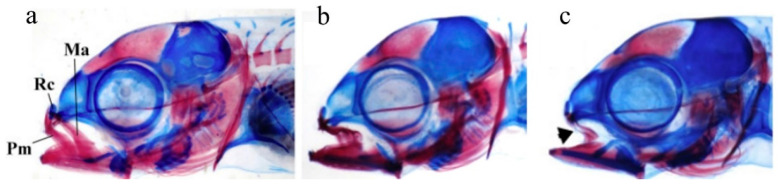
Stereomicrograph of different abnormality types of the maxillaries and premaxillaries in gilthead seabream. (**a**) Normal anatomy of maxillaries (Ma), premaxilaries (PM), and rostral cartilage (Rc), in a seabream larva (9.5 mm TL), (**b**) size reduction in the premaxillaries; (**c**) narrowing of maxillaries (arrow) and complete absence of the premaxillaries. Adapted from Fragkoulis et al., 2018 [44].

Additionally, jaw abnormalities have been frequently reported to develop in reared finfish, and the gilthead seabream is no exception. Those abnormalities have several different types, including size reduction and deformity of the maxillaries and premaxillaries (Figure 4). Different jaw skeletal elements, including Meckel’s cartilage, dentaries, articulars, pre-maxillaries, maxillaries, ethmoid, vomer, and palatine, are involved in the abnormal phenotypes generating a very complex anatomy of abnormalities [19,34,44]. Fish with head deformities swam normally; however, their growth may have been affected as is the case in all other forms of abnormalities [34,44].

### 3.3. Spinal Deformity

**Figure 5:**
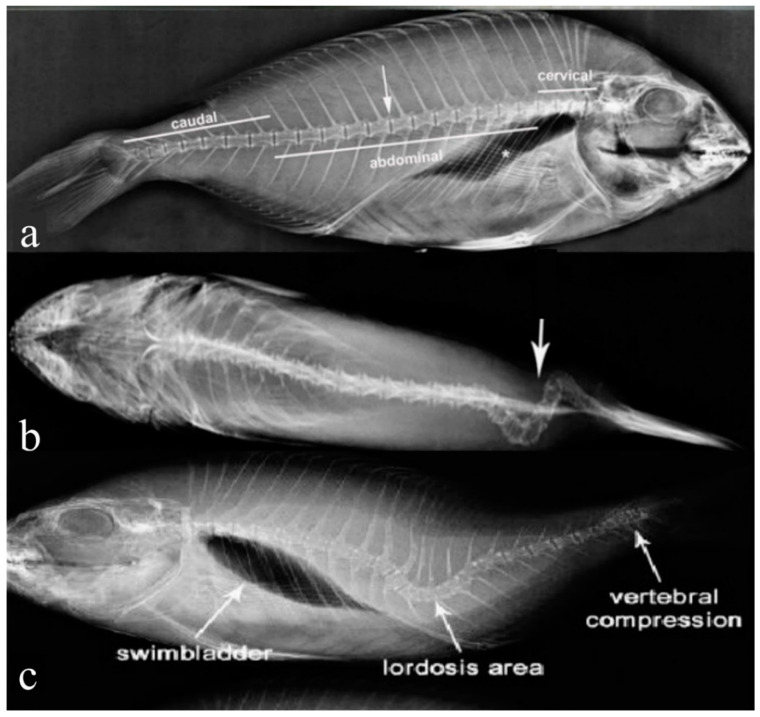
X-ray of gilthead seabream with different forms of spinal deformities. (**a**) Normal anatomy of cervical, abdominal, and caudal regions of the vertebral column. Inter-vertebrae space is highlighted with an arrow, and swim bladder is indicated with a star; (**b**) apical X-ray of seabream caudal region with scoliosis (highlighted with arrows); (**c**) X-ray of seabream with coexisting two main deformities of the vertebral column lordosis and vertebral compression. (**a**,**b**) Are adapted from Boursiaki et al., 2019 [48], while (**c**) is adapted from Berellis et al., 2015 [49].

In the gilthead seabream production industry, vertebral deformities are frequent abnormalities affecting between 27% and 50.3% in seabream larvae, 17.2% of post larvae, and 5% of adults [45,46]. Three main types of deformities exist, differentiated by the direction of their curvature. Scoliosis is defined as an aberrant lateral curvature of the vertebral column, detected either by dorsal or ventral examination (Figure 5a,b). Lordosis, however, is an abnormal ventral bend of the vertebral column accompanied by atypical calcification of the afflicted vertebrae compared to the normal anatomy of the vertebral column (Figure 5a,c), and finally, kyphosis is a dorsal curvature [46,47].

**Figure 6:**
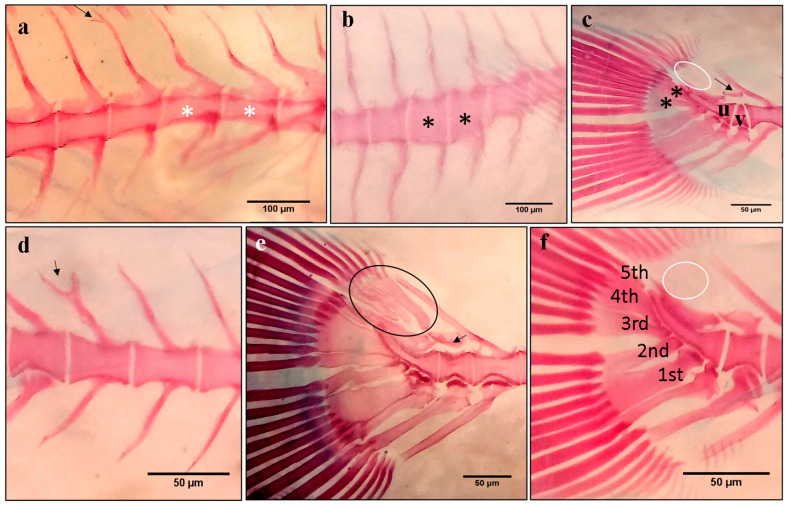
Typical forms of skeletal abnormalities in gilthead seabream larvae from the three groups of larvae at 53 DPH. (**a**) Rectangular slender vertebral body (white asterisks), and bifurcated neural spine (arrow); (**b**) cubic thick vertebral body (black asterisks); (**c**) the last caudal vertebra (v), preceding the urostyle (u), is triangular-shaped with detached neural spine (arrow). The absence of the three epurals (white circle), lack of the fifth hypural, and fusion of the third and fourth hypural (black asteriks); (**d**) bifurcated neural spine (black arrow); (**e**) detached neural spine (arrow). The circle indicates a normal epural arrangement; (**f**) normal hypural arrangement (1st to 5th hypurals) and absence of the three epurals (white circle). Scale bars (**a**,**b**): 100 µm, and (**c**–**f**): 50 µm. The figure was adapted from Mhalhel et al. [12].

Axis deformations are considered among the predominant types of spinal deformities besides the compression and fusion of vertebral bodies and some phenotypes of lack or extra-formation of the different vertebral elements [45,50,51]. The spinal column deformities vary with the degree of deformity and in the number of flexions of the vertebral column, resulting in a continuous distribution of the external morphology ranging from insignificant to severe body-shape alterations [52]. Different studies recorded thirty-nine types of skeletal deformities attributed to several malformation-altered vertebrae, including rectangular slender vertebral body (Figure 6a), cubic thick vertebral body (Figure 6b), and triangular-shaped vertebrae (Figure 6c) [12,46,51]. These basic deformities have been reported to develop in a solitary manner or in various combinations of different types, e.g., lordosis and kyphosis in the saddleback syndrome [47] or the consecutive repetition of lordosis/scoliosis/kyphosis (LSK) from head to tail, which was reported in some studies on gilthead seabream [13,46]. They first appeared at the larval stage and have been correlated with the absence of a functional swim bladder, which has been reported to be partly or totally corrected following late inflation of the swim bladder, or to other biotic and abiotic factors [49].

Fish with spinal deformities either swim upside-down or sideward, and their growth is slow compared to normal fish [16].

### 3.4. Caudal Fin Deformity

**Figure 7:**
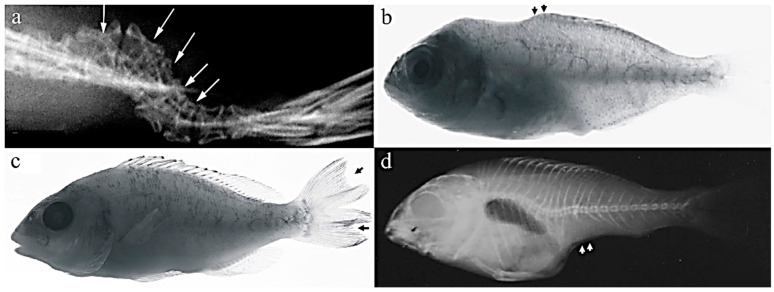
Caudal-fin deformities in gilthead seabream. (**a**) Lateral twisting of the whole caudal complex, (**b**) incomplete formation of dorsal fin in juvenile *S. aurata*, (**c**) duplication of caudal fin, and (**d**) complete lack of anal fin in juvenile *S. aurata*. The figure was adapted from Koumoundouros et al. [51].

Caudal fin abnormalities have been reported to develop in reared fish, and their prevalence in gilthead seabream production ranged between 25 and 65% [38,53]. Severity degrees show a wild variety of phenotypes, ranging from the lack of rays to the lateral twisting of the whole caudal complex (Figure 7a) or the duplication of the caudal fin (Figure 7c) [51,53,54], beside the bifurcation of the neural spine (Figure 6a,d) and detached neural and hemal spine (Figure 6c,e). Thus, all the elements of the caudal region (epurals, hypurals and parahypurals, neural arch, and the uroneural and vertebra centra) are implicated in the abnormalities [53].

The incomplete development of both dorsal and anal fins was reported to be one of the most severe deformities (saddleback syndrome) in the gilthead seabream and other fish species (Figure 7b,d) [51]. Those deformities were highly correlated to posterior notochord deformities, mainly the lack of upper lepidotrichia and dermatotrichia at the pre-flexion phase [51].

The existing literature on the causative factors of these abnormalities mostly focuses on the effects of the rearing environment, including temperature, nutrition, dietary levels of vitamin A, and the genetic basis [38,51,54].

### 3.5. Body Shape Deformities

The body shape is the main quality trait of reared fish, especially those sold as a whole, like the gilthead seabream. Several studies proved that shape deviations from the typical pattern are usually affected by all the previously detailed skeletal abnormalities [51,55]. However, the shape differentiations are very common even in subjects without skeleton deformities, as a direct result of the different rearing conditions and the genotype [51,55]. In fact, canonical variate analysis revealed that both rearing methodologies and the origin of the fish, namely wild or reared, significantly affect the body shape of seabream during the on-growing phase [56].

## 4. Cause of Deformities

Deformities are one of the most recurrent biological problems affecting finfish aquaculture, defined as the common abnormal transformations of normal skeletal structures into abnormal structures different from the normal prototype in both wild and cultured fish populations; however, their frequencies are greater in hatchery populations [14,51]. The high incidence of skeletal deformities found in farmed fish can be explained by the maximized fish survival due to the absence of predators and the high availability of food. Technical errors or knowledge gaps in some critical segments of the rearing process were also suspected [23,53]. Available evidence suggests that those abnormalities appear in the early stages of development during the embryonic and larval stages of life, long before they can be externally visible [16]. However, the etiology of these syndromes is not yet well understood [16].

Osteogenesis is ensured by a complex set of regulated molecular pathways involving signaling molecules, transcription factors, and extracellular matrix (ECM) constituents, among which alkaline phosphatase (ALP) or non-collagenous proteins such as the matrix Gla protein (MGP) or osteocalcin (OC) [23]. Thus, the perturbation of those factors implicated in the control of bone development and homeostasis, beside the incapacity of the self-regulating process to compensate for the stressful environmental conditions, induce the disruption of skeletogenesis and lead to the appearance of skeletal deformities [14,23].

Different studies suggest that unfavorable environmental biotic, abiotic disturbances, nutritional imbalances, presence of xenobiotic substances and/or genetic disorders, and unsuitable rearing conditions are the most probable causative agents of deformities in reared fish, to which we can add traumatic injury and parasite infections [14,16,23,35].

Several studies have listed the different abnormalities affecting the skeletal structures in cultured seabream and have reported the effects of some rearing conditions (temperature, light) or substances (vitamins) on the prevalence of bone abnormalities. However, etiological knowledge is still insufficient for elaborating Cartesian experimental hypotheses concerning the predisposing causes of the apparition and the underlying processes behind abnormalities since this is considered a multifactorial problem [14,19].

Various fish pathogens, including viruses, bacteria, and parasites, can potentially trigger skeletal deformities [1]. For example, the infectious hematopoietic necrosis virus in rainbow trout significantly increased the incidence of spinal deformities [2]. Infection with Streptococcus agalactiae in Nile tilapia may lead to deformities affecting the cephalic region, the vertebral column, as well as the fin and tails. These skeletal deformities induced by streptococcosis infections could be explained by the desquamation of gastrointestinal mucosa, thereby hindering the absorption of dietary nutrients [3]. Moreover, pathogenic agents such as Myxobolus sandrae Reuss and Myxobolus cerebralis have been reported as responsible for vertebral column deformities, and severe spinal cord lesions in perch, rainbow trout, the European chub, the roach, and the common bream [4,5].

Although a wide range of fish pathogens and their potential impact on skeletal health have been detected in many fish species, to the best of the authors’ knowledge, no cases of bone deformities associated with fish pathogens have been found in the gilthead seabream.

The environmental contaminants were generally controlled under rearing conditions which can explain the rarity of studies reporting its causality of skeletal abnormalities in reared gilthead seabream.

The only study reporting the effect of cademium on skeletogenisis was that of Sassi and his co-authors, where the concentrations of 5 and 10 mg/L of cadimium down-regulated the transcript levels of osteocalcin and disrupted bone mineralization [6].

Finally, stress arising during larvae handling has also been reported as a possible cause of notochord distortions [7].

## 5. Effect of Deformities on Production and Welfare

The welfare of the gilthead seabream is a matter of concern due to the potential far-reaching consequences of skeletal abnormalities. Such abnormalities in fish can result in various adverse effects on their regular physiological functions. These include impacts on buoyancy, swimming ability, respiration, conversion efficiency, growth rate, production costs, and an increased susceptibility to stressors and diseases. These issues can impede a fish’s ability to evade predators and overcome environmental challenges, posing a significant threat to their well-being [11,23,26,46].

Deviations in the cranial skeleton, specifically mouth deformities, can give rise to two primary challenges. Firstly, it can hinder the fish’s ability to move their jaw and ingest food, potentially leading to starvation. This impediment not only hampers normal growth and development but also hinders these fish from reaching their full potential size and weight, thereby affecting their overall health and survival rates [57,58]. The second major issue faced by these fish is the compromised respiratory efficiency, resulting from a reduced capacity to utilize buccal–opercular pumping for proper gill ventilation. This phenomenon has been observed in fish with lower jaw skeletal abnormalities and pug-headedness [20]. Fish with such mouth deformities become more susceptible to hypoxic stress and may need to swim more to ensure adequate water flow over their gills, further compounded by high stocking densities that can hinder their swimming abilities. Consequently, it is likely that abnormal behaviors play a role in explaining the occurrence of mechanical damage to the mouths of cultured fish [43,57].

To the best of the authors’ knowledge, there is no existing documentation on how fin damage impacts gilthead seabream growth performance and survival. Moreover, the potential consequences of fin damage, which could be inferred from tagging studies examining the effects of fin clipping on growth and survival, were constrained due to the scarcity and age of these studies. In his study conducted in 1975, Lasserre found that pelvic fin clipping had no impact on fingerling growth. Similar observations were noted in studies involving various species. Indeed, the removal of a single fin does not reduce growth but does reduce survival in the rainbow trout [59]. Furthermore, fish with multiple fin excisions exhibit a lower survival rate compared to those with a single fin removed [60]. In a study by Zymonas (2006), the growth performance of bull trout *Salvelinus confluentus* remained unaffected even after the removal of three pelvic fin rays [61].

It is conceivable that fin damage may compromise fin function, potentially negatively impacting a fish’s ability to maintain its posture and swim effectively or compete for food, leading to a loss of weight and reduced growth. However, the outcome may vary depending on the severity and type of fin damage, whether it involves erosion or splitting [57,58]. Moreover, spinal and swim bladder issues are reported, as well, to have a significant impact on the gilthead seabream’s swimming performance [62].

A concise and well-researched review of the potential effect of injuries and abnormalities upon fish welfare was carried out, based on a number of associative inferences, by Branson and his team in 2008 and Noble and his co-authors in 2012 [57,58]. Both studies underscored the scarcity of recent, quantified investigations directly assessing the consequences of deformities on fish welfare. These observations emphasize the critical need for further investigation into these effects on fish welfare given the well-established evidence that fish are capable of experiencing pain and suffering [63].

Understanding and addressing the impact of skeletal deformities on gilthead seabream welfare is essential not only from an ethical standpoint but also for the sustainability of aquaculture practices and the health of marine ecosystems.

## 6. The Genetic Component of Bone Deformities

**Figure 8:**
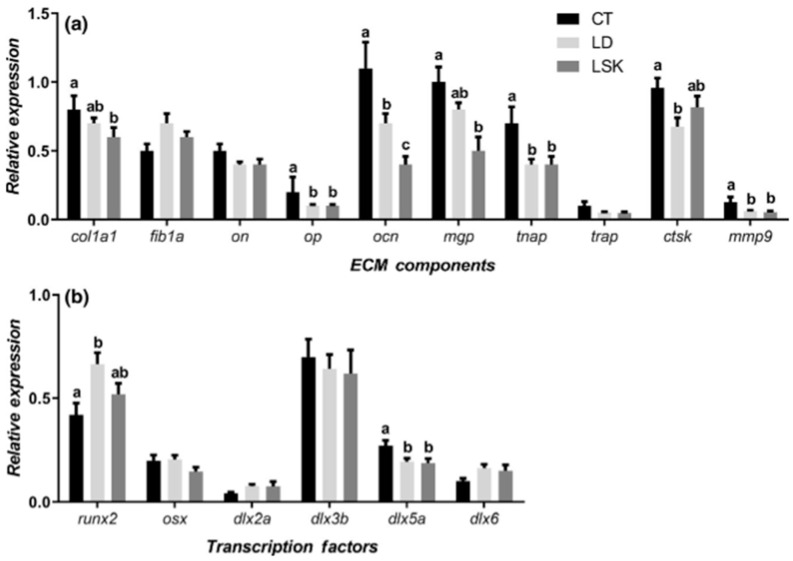
Relative gene expression of (**a**) ECM components and (**b**) transcription factors in gilthead seabream vertebral column fragments of control animals (CT) or specimens with lordosis (LD) or lordosis–scoliosis–kyphosis (LSK). Results are shown as the mean ± SEM. Distinct letters denote statistically significant differences among groups (*p* < 0.05). The figure was adapted from Riera-Heredia et al. [64].

In a study conducted on reared gilthead seabream, Riera-Heredia and her co-authors investigated the extracellular matrix components’ gene expression and bone formation and regeneration transcription factors in fish with the different skeletal anomalies (lordosis (LD), lordosis–scoliosis–kyphosis (LSK), opercular, dental, or jaw abnormalities), compared to control (CT) specimens [64]. The finding of this study revealed a potential association between the presence of LD and LSK and the significant down-regulation of genes involved in the maturation of the osteoblasts and matrix mineralization, namely collagen type 1-alpha (col1a1), osteopontin (OP), osteocalcin (OCN), matrix Gla protein (mgp), and tissue non-specific alkaline phosphatase (tnap), as well as cathepsin K (ctsk) and matrix metalloproteinase 9 (mmp9), taking part in bone resorption compared to the CT group’s fish. Contrarily, runx2, the key osteogenic transcription factor, exhibited an up-regulation within the malformed vertebra. This suggests an impaired mesenchymal stem cell determination toward the osteo-blastic lineage (Figure 8) [64].

In our study conducted on gilthead seabream larvae, we investigated bone and skeletal muscle-specific gene expression, parathyroid hormone-related protein-coding gene (PTHrP), and myosin light chain 2 (mlc2), respectively, during the larval and post-larval stages [12]. The marker of muscle development and growth, mlc2, one of the four light chains of the myosine, exerts its regulatory role in binding calcium [65]. PTHrP is a calcium regulatory factor in gilthead seabream that has a crucial function in several physiological and biochemical processes, including calcium mobilization from internal sources (bone and scales) and via calcium uptake from water and diet [66], as well as tissue differentiation and proliferation [67].

We have reported an association between abnormally developing bones and a high expression of PTHrP on Sparus aurata [12], and the same effect was assigned to PTHrP up-regulation in the immature seabream’s systrophic spine by Ingleton [68]. Myosin, a key component of striated muscle contributing to muscle contraction, consists of two heavy chains (MHCs) and four light chains (MLCs). Myosin light chain-2 is a sarcomeric protein expressed in the white muscle of gilthead seabream as two isoforms, mlc2a and mlc2b. Myosin light chain 2a predominates the early larval stages, marking new fiber development at the tissue level. In our study, the inverse dose-dependent relationship of mlc2 downregulation and exogenous MEL concentration during the treatment period was associated with the negative allometric growth pattern by MEL. Our results are in accordance with the significant weight increase registered in Atlantic salmon implanted with melatonin and contrasting the body weight and growth rate reduction induced by implants or injections in both trout and goldfish [69,70,71].

In their study conducted in 2022, Gerogiou and his co-authors examined the potential associations between mRNA levels of cathepsin D, cathepsin Z, cyclin-A2, and glucocorticoid receptor and the occurrence of skeletal abnormalities during the larval period [72]. Their findings demonstrated that relatively high levels of cyclin-A2 and glucocorticoid receptor were found to be associated with the early prediction of egg quality but not correlated with the occurrence of skeletal abnormalities [72].

## 7. Mitigating Skeletal Abnormalities in Gilthead Seabream: Strategies for a Sustainable Aquaculture

The management and prevention of skeletal abnormalities play a crucial role in guaranteeing the sustainability of aquaculture production. The development of skeletal disorders is associated with a complex interplay of factors, including nutrition, environmental, and genetic factors [38]. In order to address and mitigate the adverse effects of skeletal abnormalities in the gilthead seabream, it is crucial to understand the environmental preferences and nutritional needs specific to each stage of the species, along with comprehending the ontogeny of the skeletogenesis and the characteristics of different deformities [38]. By gaining this understanding, adjustments can be made in feeding methods, rearing conditions, and selective breeding programs to address these issues effectively.

The impact of nutrition on bone development and remodeling in the gilthead seabream has been the subject of several studies, with the available information being somewhat fragmentary, depending on the specific nutrients in consideration [73]. Recent advances in formulating starter diets for larvae and enriching emulsions for live prey have revealed various essential nutrients, particularly vitamins, lipids, and minerals, crucial in normal skeletogenesis. Consequently, the improper balance or inadequate supply of these nutrients in the diet may develop skeletal deformities [74].

Among the fat-soluble vitamins, vitamins A, D, and E have been the subjects of numerous studies, each aiming to unveil their specific impacts on the development of seabream bones. Prior studies have conclusively highlighted the crucial role of vitamin A, as demonstrated by Fernández and his co-authors [38,75], illuminating its significance in bone health and underlining the risk of the alteration of bone homeostasis by hypervitaminosis A, accelerating bone mineralization, and inducing vertebral compression or fusion in juvenile fish [75]. Furthermore, the works of Dominguez and Sivagurunathan in 2021 and 2022 have underscored the potential role of vitamin D in averting skeletal irregularities in seabream [76,77,78]. Similarly, Izquierdo and Saleh’s studies delved into the ramifications of vitamin E within the context of bone deformities [37,79].

Research on the dietary levels of vitamin K and its influence on the skeletal development of seabream has been sparse, with the most recent investigation carried out on juvenile specimens by Dominguez in 2022 and Sivagurunathan and his co-authors in 2023 [80,81].

Implementing a nutrient-rich diet in essential nutrients, especially calcium and phosphorus, was suggested as a promoter for proper bone development, and balanced diets in these minerals can help prevent skeletal abnormalities [48].

Furthermore, insufficient DHA levels appear to correlate with a heightened prevalence of lordosis and kyphosis and a reduced count of mineralized vertebrae. Likewise, increased dietary DHA content led to an escalation in deformities within cranial endochondral bones and axial skeletal hemal and neural arches. Consequently, a moderate dosage of 3.20 ± 0.15% of the body weight was determined to be the optimal choice [37].

While there is a rich bibliography on the nutritional requirements to address bone deformities, there has been a noted absence of investigations into the impact of the gut microbiome on nutrient utilization and skeletal development in the gilthead seabream. Thus, the potential benefits of probiotics and dietary interventions for enhancing bone health in the gilthead seabream remain unexplored. This highlights a significant research gap in our understanding of these crucial aspects of gilthead seabream biology.

Optimizing the rearing environment has a profound influence on skeletal health. This entails the accurate maintenance of rigorous water quality parameters, including temperature [40,82], oxygen levels [20], as well as stocking densities [2]. This careful maintenance creates an ideal habitat and plays a pivotal role in stress reduction.

In addition, another crucial aspect of this comprehensive strategy involves the management of lighting and photoperiod conditions [12,83].

Even though controlling the environmental parameters, such as biotic and abiotic, physiological, xenobiotic, nutritional, and rearing factors, can reduce the prevalence of deformities, it cannot wholly eliminate the abnormalities [84]. Therefore, employing animal breeding control and genetic selection could be a good strategy to decrease these deformities’ prevalence permanently.

Intensive selective breeding programs were recognized as an effective strategy adopted to overcome bone deformity issues for gilthead seabream mariculture, improving the growth performance [85] and morphology [73,86] by preserving or neglecting the DNA markers tightly linked to traits-affecting genes provided by the genomic region closely linked to major effect genes known as quantitative trait loci (QTL).

Several QTL associated with traits of commercial importance in the gilthead seabream were identified using microsatellite markers. These QTL were related to various aspects, such as growth and morphometric traits. Several QTL related to growth and morphometric traits were highlighted in multiple studies by Loukovitis et al. (2011, 2012, 2013) [87,88,89], and one significant QTL for morphometric traits was detected by Boulton et al. (2011) [90].

In a study by Negrín-Báez and co-authors [91], they reported the identification of seven quantitative trait loci (QTL) associated with frequent lordosis, vertebral fusion, and jaw abnormalities. When it comes to vertebral fusion, the investigation revealed the presence of one significant QTL, along with two suggestive QTL. For lordosis, one significant QTL at the chromosome and genome-wide levels and one suggestive QTL were detected. Lastly, in relation to jaw deformities, the investigators identified one significant QTL and one suggestive QTL [91]. The three significant QTL, however, were detected using a unique full-sibling family; thus, they could not be representative for other gilthead seabream families.

Implementing a genetic selection program to identify and breed non-deformed individuals has the potential to significantly reduce the occurrence and severity of skeletal abnormalities, improve overall fish health, and enhance the sustainability of aquaculture operations.

## 8. Perspective for Investigating Bone Defects

In order to improve cost-efficiency for farmed gilthead seabream, fast and early recognition of developing abnormalities is of great importance for fish farmers since abnormal subjects, with adversely affected growth rates and reduced survival rates, will compete for food and space with healthy fish [57]. A careful examination should be conducted on both sides of each fish. When the percentage of deformities in a given fish population exceeds the quality standards, the deformed animals should be sorted out. The only effective technique to remove such fry is to sort them by hand [35].

For this reason, various procedures have been applied as simple and rapid diagnostic tools for studying skeletal deformities, abnormal growth in different skeletal structures, as well as the unusual swimming behavior revealing abnormal development in fish. The visual examinations, including the analysis of geometric morphometrics, stereoscopic observations, double staining, computer tomography, and soft X-rays, allow for the description of shape variation in growing and transforming fish and the effect of rearing conditions on the body shape [14,35]. The histological procedures, bone mineral density, and calcium content measure, as well as quantifying metabolic bone disease markers, namely bone-specific alkaline phosphatase, are other valuable tools for providing elementary knowledge on bone formation and the structural and physiological changes occurring in deformed skeletal structures [14].

All the above-mentioned analyses on morphology allowed for a quantitative and qualitative analysis of the nature of osteological aberrations in fish and the different degrees of shape changes in their external morphology.

## 9. Gilthead Seabream as a Model for Understanding Skeletogenesis

Different studies on fish have recognized teleost as a suitable vertebrate model for understanding vertebrate development and metabolism, particularly skeletogenesis in lower and higher vertebrates, for comparative and evolutionary purposes [31,92,93,94,95,96].

The resemblance of physiological processes from fish to mammals and the presence of fish orthologs for most mammalian gene regulatory networks for skeletogenesis are among the traits that contributed to the recent interest in fish models.

Zebrafish and medaka are the predominant fish models in bone biomedical research. They are used as an in vivo model of several human bone conditions, including holospondyly osteogenesis, craniofacial dysplasia, and imperfecta, and for the exploration of the role played by the different genes in bone formation, maintenance, skeletal development, and abnormalities [97,98]. Despite their potential for translational research, it is important to note that both species may not serve as ideal models for aquaculture in marine fish under specific conditions. In fact, aside from their inability to encompass species with acellular bone structures, they fail to meet the abiotic requirements essential for marine fish.

The skeletal system of the gilthead seabream is categorized as acellular since it is generated and maintained by chondrocytes, osteoblasts, and osteoclasts, yet it does not contain osteocytes within its calcified extracellular matrix [99].

The study of the transcription factors involved in the formation and turnover of bone on gilthead seabream gill arch and vertebra has revealed homologs of many mammalian skeleton-related transcripts including osteoblast differentiation transcription factors (Runx2/Cbfa1, osterix/Sp7), or components of the extracellular matrix that regulate mineral deposition such as osteonectin/SPARC, osteopontin/Spp1 and osteocalcin/BGP [100]. Moreover, the BMP2 mRNA expression pattern detected in the gilthead seabream mesenchymal stem cells cultures described by Riera-Heredia and his co-authors was found to be coherent with the BMP2 association with the process of extracellular matrix mineralization and bone nodule formation during mesenchymal stem cell differentiation in mammals [101].

Furthermore, gilthead seabream larvae, like other marine fish, hatch much earlier in their development (39.41 h post fertilization) than the confirmed teleost model zebrafish (approximately 72 h post fertilization) or other vertebrates, making it an attractive model to study the influence of several nutrient deficits (vitamin, calcium, and phosphorus…), and genetic factors in morphogenesis and skeletogenesis during early larval development [28,102]. It exhibits indeterminate growth, in contrast to other vertebrate groups and zebrafish, which means that they can grow in length and weight beyond the maturation stage for as long as they live [103], allowing researchers to observe bone growth and regeneration over an extended period, providing insights into bone remodeling processes. This characteristic allows researchers to study aquaculture fish adaptability to the different rearing conditions and bones’ differential responses to those factors.

In recent decades, important biochemical, molecular, and cellular tools have been developed [104]. These tools include several genomic sequences, large collections of expressed sequence tags (EST) for several fish species, including the gilthead seabream [105,106], European seabass [106], Atlantic salmon [107], high-density DNA microarray platforms to explore these EST collections [108,109], and Agilent SurePrint™ Technology oligo-array, namely for the gilthead seabream.

Various fish bone-derived cell lines are available for investigating tissue mineralization mechanisms [110,111]. Indeed, in 2004, Pombinho et al. developed two bone-derived cell lines, VSa13 and VSa16, from the marine teleost gilthead seabream [112]. The two cell lines are particularly interesting with a pre-chondrocyte and pre-osteoblast phenotype, respectively. Indeed, they have a differential capacity of mineralizing their extracellular matrix with different degrees of mineral deposition, different levels of alkaline phosphatase activity, and matrix gla protein (MGP), osteopontin (SPP1), osteocalcin (OC), bone morphogenetic protein-2 (BMP-2) mineralogenic genes’ expression, as well as a distinct susceptibility to mineralogenic or osteogenesis inhibitors [30,112]. Therefore, they have been used to identify key mineralogenic genes differentially expressed during in vitro mineralization on the different bone cell types [30].

While the gilthead seabream can be a valuable model for certain aspects of bone research, there are potential limitations and negative points to consider when using this species, mostly its limited genetic resources. Indeed, compared to more established model organisms like zebrafish or mice, the gilthead seabream has limited available genetic and molecular resources. It lacks well-established mutant lines that allow researchers to study specific genes and their roles in bone development.

Furthermore, the research community dedicated to the gilthead seabream is smaller when compared to the widespread utilization of canonical models. This discrepancy results in fewer available protocols and research tools.

Finally, maintaining gilthead seabream in a research facility can entail higher costs and logistic challenges than other model organisms.

## 10. Conclusions

Our review provides an overview of skeletal development and anomalies in the gilthead seabream, highlighting the genetic, environmental, and nutritional factors contributing to bone deformities and emphasizing the potential of the gilthead seabream as a model organism for understanding bone morphogenesis in both aquaculture and translational biological research.

Future research should explore the potential of the microbiome on nutrient utilization and indirectly on skeletal development. To address this complex issue, a collaboration involving aquaculture scientists, geneticists, nutritionists, and environmental specialists could offer holistic solutions to mitigate the impact of skeletal abnormalities.

Developing databases and implementing big data analytics to integrate information from diverse studies could uncover correlations that may not be evident in individual experiments.

Pursuing these research directions could enhance our understanding of skeletal development and anomalies in the gilthead seabream, optimize aquaculture practices, and contribute to the broader knowledge related to bone abnormalities, ultimately benefiting both the aquaculture industry and translational biological research.

## Figures and Tables

**Table 1:** VSa13 cells’ genes regulated during mineralization of (FC_M_) versus mineralization + vanadate (FC_MV_).

Identified Genes	GO: Biological Processes/Molecular Function	FCM/FCMV Score
Photoreceptor outer segment all-trans retinol dehydrogenase [IPIAcc:IPI00024598]	metabolic process/oxidoreductase activity	240.1
AMBP protein precursor [Uniprot Acc:P02760]	-/-	131.5
Ependymin related protein-1 precursor [IPIAcc:IPI00554718]	cell-matrix adhesion/calcium ion binding	111.8
Xaa-Pro aminopeptidase 2 precursor [Uniprot Acc:O43895]	proteolysis, creatine metabolic process/metalloexopeptidase activity, hydrolase activity, creatinase activity	110.2
Microtubule-associated proteins 1A/1B light chain 3C precursor [Uniprot Acc:Q9BXW4]	-/-	89.63
Cell death activator CIDE-3 [Uniprot Acc:Q96AQ7]	apoptosis/protein binding	45.71
PREDICTED: hypothetical protein [Danio rerio]	pore complex biogenesis/channel activity	35.20
Hypothetical protein LOC447879 [RefSeq_peptideAcc:NP_001004618]	integrin-mediated signaling pathway/-	32.64
Granulocyte-macrophage colony-stimulating factor receptor alpha chain precursor [Uniprot Acc:P15509]	-/-	29.83
IgGFc-binding protein precursor [Uniprot Acc:Q9Y6R7]	cell adhesion/-	23.12
Hydroxyacid oxidase 2 [Uniprot Acc:Q9NYQ3]	metabolic process, electron transport/oxidoreductase activity	21.38
Phosphatase and actin regulator 4 isoform 1 [RefSeq_peptideAcc:NP_001041648]	-/-	18.08
Ependymin-related protein-1 precursor [IPIAcc:IPI00554718]	cell–matrix adhesion/calcium ion binding	18.02
Glucose-6-phosphate 1-dehydrogenase [Uniprot Acc:P11413]	glucose metabolic process/glucose-6-phosphate dehydrogenase activity	16.05
Glutamate–cysteine ligase catalytic subunit [Uniprot Acc:P48506]	-/-	16.04
Complement factor I precursor [Uniprot Acc:P05156]	proteolysis/catalytic activity, serine-type endopeptidase activity, hydrolase activity, scavenger receptor activity	15.91
Hypothetical protein LOC406276 [RefSeq_peptideAcc:NP_998168]	-/calcium ion binding	12.54
Hypothetical protein LOC336637 [RefSeq_peptideAcc:NP_956317]	cell redox homeostasis/-	12.17
Actin filament-associated protein 1-like 2. [Uniprot Acc:Q8N4X5]	-/-	11.79
Transcribed locus, weakly similar to NP_175293.1 [UniGeneAcc:Tru.931]	metabolic process/methyltransferase activity	11.69
Ornithine carbamoyltransferase, mitochondrial precursor [Uniprot Acc:P00480]	-/ornithine carbamoyltransferase activity, amino acid binding	11.38
CDNA FLJ31025 fis, clone HLUNG2000501. [Uniprot/SPTREMBLAcc:Q96ND9]	-/-	11.25
Stromal cell-derived factor 1 precursor [Uniprot Acc:P48061]	-/-	10.36
ADM precursor [Uniprot Acc:P35318]	-/-	10.10
Beta-2-glycoprotein 1 precursor [Uniprot Acc:P02749]	-/-	10.05
Signal peptide, CUB and EGF-like domain-containing protein 2 precursor [Uniprot Acc:Q9NQ36]	-/calcium ion binding	78.01
Actin, alpha skeletal muscle 1. [Uniprot Acc:P68140]	-/structural molecule activity, ATP binding, protein binding	38.39
Inter-alpha globulIn InhIbItor H3 [IPIAcc:IPI00028413]	hyaluronan metabolic process/serine-type endopeptidase inhibitor activity	24.67
Hypothetical protein LOC553753 [RefSeq_peptideAcc:NP_001018560]	-/protein binding	21.14
Tenascin precursor [Uniprot Acc:P24821]	signal transduction/receptor binding	20.56
Prolargin precursor [Uniprot Acc:P51888]	-/-	18.89
Eukaryotic translation initiation factor 4E-1A-binding protein [Uniprot Acc:Q98TT6]	negative regulation of translational initiation/eukaryotic initiation factor 4E binding	12.02
WNT1-inducible-signaling pathway protein 1 precursor [Uniprot Acc:O95388]	regulation of cell growth/insulin-like growth factor binding	10.77

GO classification was subdivided into biological processes (BP) and molecular function (MF).

## Data Availability

Not applicable.
